# A case of pediatric rosacea—Skin and ocular involvement

**DOI:** 10.1002/ccr3.2482

**Published:** 2019-11-01

**Authors:** Xue Ting Ooi, Kong Bing Tan, Nisha Chandran

**Affiliations:** ^1^ Department of Medicine University Medicine Cluster National University Hospital Singapore City Singapore; ^2^ Department of Pathology Yong Loo Lin School of Medicine National University Health System Singapore City Singapore; ^3^ Division of Dermatology University Medicine Cluster National University Hospital Singapore City Singapore

**Keywords:** inflammatory skin disease, ocular rosacea, pediatric, rosacea

## Abstract

Childhood rosacea presents a diagnostic difficulty due to the lack of diagnostic criteria and potential mimics. Ocular involvement is a frequent complication of rosacea in children and may appear before cutaneous findings. It is important for clinicians to be aware of these and to screen patients appropriately in order for timely treatment to be instituted.

## Graphical abstract

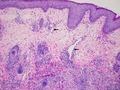

Rosacea is a chronic inflammatory skin disease characterized by flushing, erythema, telangiectasia, papules, and pustules in the central convex areas of the face. A phenotype‐based approach to rosacea diagnosis and classification is an update to the previous subtype classification system.^1^ Rosacea is most commonly seen in middle‐aged women and is rare in the pediatric population. Phymatous change is not seen in the pediatric population. However, it is hypothesized that childhood rosacea is probably underreported due to the lack of diagnostic criteria in this age group.^2^

An Indian male was first seen at the age of 9 years for a 4‐year history of asymptomatic facial rash predominantly over the forehead and bilateral cheeks. On examination, there were multiple monomorphic glistening papules on the forehead and cheeks (Figures 1 and 2); a diagnosis of lichen nitidus was made. He was treated with topical calcineurin inhibitors and mild potency topical steroids. Half a year later, the patient developed erythematous papules coalescent into infiltrated erythematous plaques over bilateral cheeks. These were distinct from the lesions typical of lichen nitidus. An initial diagnosis of steroid‐induced rosacea was made. The erythematous lesions resolved with 6‐week course of oral erythromycin and cessation of topical steroids. The condition flared again 4 months later, with a particular history of sun exposure during soccer training. A skin biopsy was performed at this point. Histology revealed a prominent dermal granulomatous infiltrate composed of aggregates of epithelioid histiocytes and multinucleated giant cells, and a heavy admixture of small lymphocytes with no definite lymphocytic atypia (Figure 3). The upper dermis showed mild dilation of postcapillary venules (Figure 4). The epidermis shows mild acanthosis. No fungi, acid‐fast bacilli, or demodex were seen with the GMS and Ziehl‐Neelsen stains. Concurrently, the patient was being treated by an ophthalmologist for noninfectious keratitis with response to steroid eye drops. A unifying diagnosis of granulomatous rosacea with ocular involvement was made.

**Figure 1:**
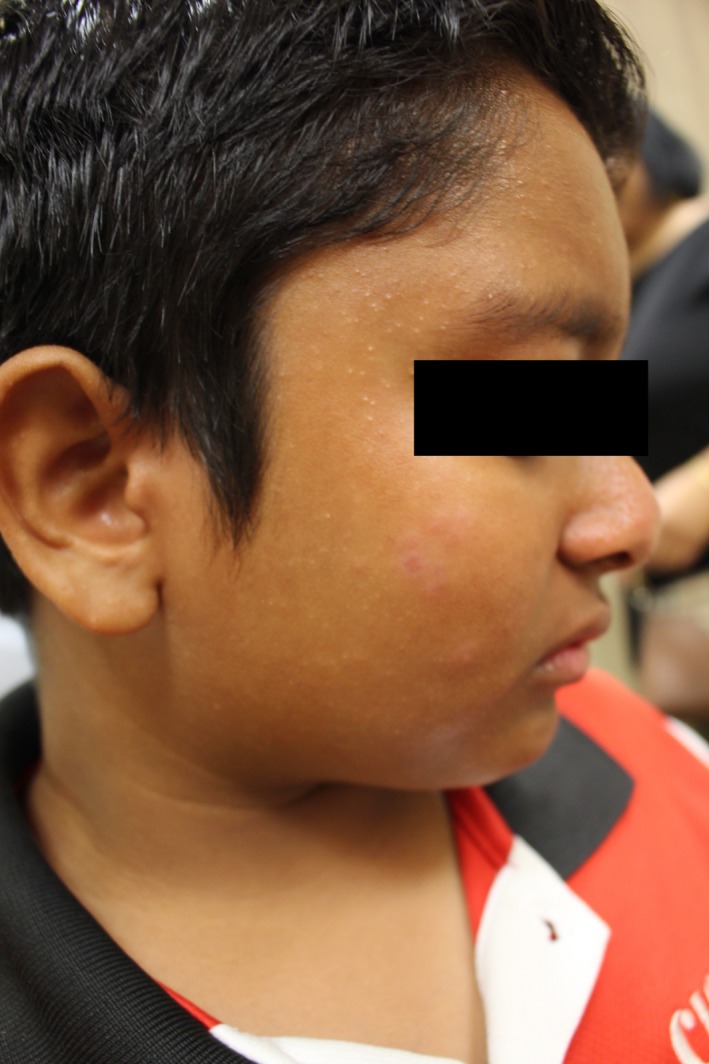
Facial rash before treatment—right cheek

**Figure 2:**
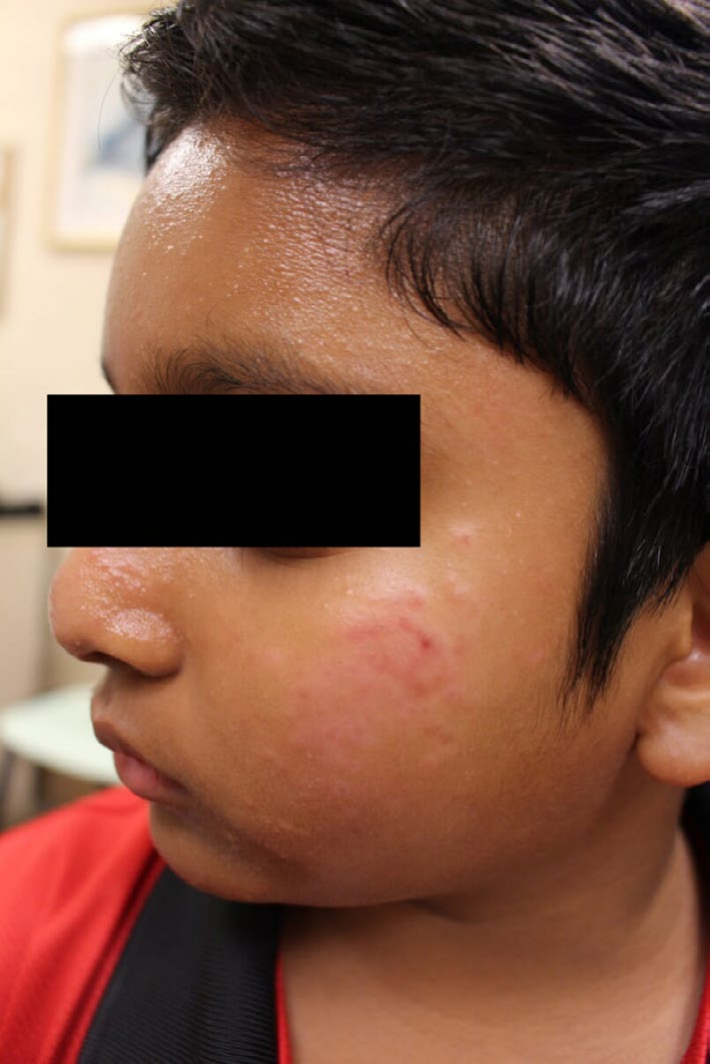
Facial rash before treatment—left cheek

**Figure 3:**
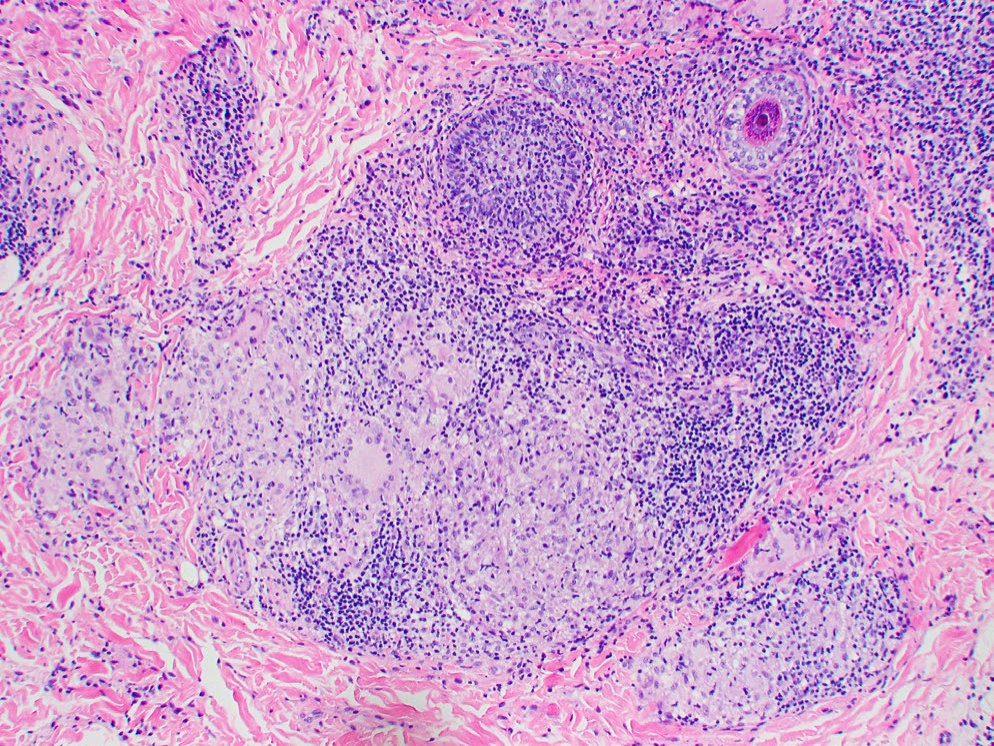
Photomicrograph of the skin biopsy shows a non‐necrotizing granulomatous and lymphocytic infiltrate in the dermis. H&E, original magnification ×100

**Figure 4:**
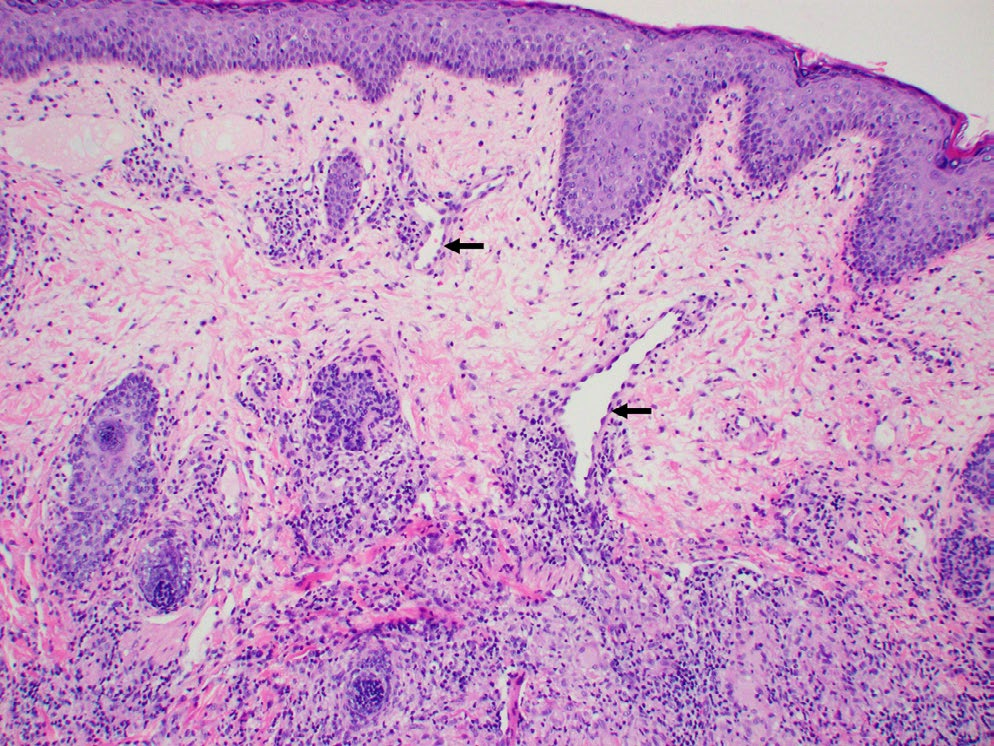
Photomicrograph showing dilated postcapillary venules (arrows) in the superficial dermis above the granulomatous infiltrate. H&E, original magnification ×100

The patient was initiated on a repeated 12‐week course of oral erythromycin with sun protection. Patient has been followed up regularly since diagnosis. Sun exposure appears to be a consistent trigger in his flares, each time occurring after outdoor activity.

Childhood rosacea presents a diagnostic difficulty due to the lack of diagnostic criteria and potential mimics. In adults, diagnosis of rosacea was previously based largely on the presence of 1 or more of the following primary features (with distribution in the central face): flushing, nontransient erythema, papules and pustules, or telangiectasia. A recent update from the global ROSacea Consensus (ROCSO) panel recommended a phenotype‐led rosacea diagnosis and classification. Under the new classification, there are two specific diagnostic phenotypes: persistent centrofacial erythema associated with periodic intensification by potential factors or phymatous changes. For patients with no diagnostic phenotype, rosacea can be diagnosed if they possess at least two major criteria which include flushing/transient erythema, nontransient erythema, telangiectasia, inflammatory papules, pustules, and ocular manifestations. Stinging sensation, edema, dry sensation, and burning sensation are minor criteria which are not necessary for diagnosis.^1^

In a study of 20 pediatric patients with rosacea, *Chamaillard* proposed that a diagnosis of rosacea in children is supported by two instead of one primary diagnostic features.^3^ Ocular involvement is a frequent complication of rosacea in adults, and it is exceptional in children.^4^ Ocular involvement in both children and adult includes blepharitis, episcleritis, keratoconjunctivitis, ocular redness, photophobia, and rarely corneal ulcers. In children, ocular signs of rosacea may appear before any cutaneous findings, thus a high level of suspicion is required for children who present with chronic ocular irritation not responding to first‐line therapy.^5^ Awareness of the clinical picture in children coupled with histological confirmation allows clinicians to better diagnose and manage pediatric patients with rosacea. A low threshold for ophthalmological screening is desired in patients with eye symptoms.

Treatment of rosacea in the pediatric population is similar to adults and it involves lifestyle modifications such as avoiding known triggers, topical, and systemic antibiotics. Prognosis varies with some children successfully tapered off systemic agents and maintained on topical therapies while others may require low‐dose systemic antibiotics. Although flares may be controlled within weeks to months, childhood rosacea tends to persist into adulthood.^6^

In conclusion, this case report highlights the importance of considering less common differentials in skin lesions that do not respond well to first‐line treatment or that are recurrent. Conditions such as rosacea may present with ophthalmic complications before the rash itself. A delay in treatment may lead to increased morbidity including visual loss.

## CONFLICT OF INTEREST

There is no conflict of interest to declare from all authors.

## AUTHOR CONTRIBUTIONS

Ooi Xue Ting: collected data and wrote the manuscript. Tan Kong Bing: provided histology slides and description for each slide. Nisha Suyien Chandran: oversee the entire manuscript writing and fine‐tune details. All authors contributed to the final version of the manuscript.

## CONSENT FOR PUBLICATION

Consent for publication has been obtained from all authors.

## CONSENT FOR PHOTOGRAPHY

Consent for photography has been obtained from patient's parent.
